# The Impact of Offensive Duration on NBA Success: A Comparative Analysis of Jordan's Chicago Bulls and Curry's Golden State Warriors

**DOI:** 10.5114/jhk/197207

**Published:** 2025-04-30

**Authors:** Mario Amatria Jiménez, Natán Andrés Cook Vaquero, Concepción Suarez-Llorca, José Antonio Pérez-Turpin

**Affiliations:** 1 Faculty of Education, Pontifical University of Salamanca, Salamanca, Spain.; 2 Department of Developmental and Educational Psychology, University of Alicante, Alicante, Spain.; 3 Department of General Didactics and Specific Didactics, University of Alicante, Alicante, Spain.

**Keywords:** game pace, game tactics, team sports, observational methodology

## Abstract

This study investigated the influence of offensive play duration on the competitive performance of two NBA teams with the most regular-season victories in the league history. Using observational methodology, differences in the length and speed of offensive actions and their corresponding shooting efficiency were examined. Results revealed significant contrasts in the play tempo, with one team favoring fast, explosive plays (Golden State Warriors) and the other adopting slower, more strategic maneuvers (Chicago Bulls). Despite these differences, both teams maintained comparable shooting success rates. This suggests that the ability to adjust the play tempo according to game circumstances and opponent characteristics is critical for maximizing offensive efficiency. Moreover, the study demonstrates that variability in possession duration is strongly linked to fatigue management and the balance between anaerobic and aerobic energy systems. This finding underscores the importance of tactical flexibility, as adjusting the pace and intensity of play can be a decisive factor in modern basketball success, where shorter, quicker, and more explosive plays are increasingly prevalent. The study concludes that dynamic time-of-possession management and adaptive efforts are key to optimizing high-level competitive performance.

## Introduction

The duration of offensive plays in basketball has long been a subject of scientific inquiry due to its direct impact on team performance (Fort-Vanmeerhaeghe et al., 2016). Offensive play duration not only affects efficiency, but also shapes the game’s tempo, player fatigue, and the ability to respond to defensive pressure (Oliver, 2011; Sampaio et al., 2010). Teams that adjust the duration of their offensive possessions based on the opponent’s characteristics and the game context tend to achieve higher levels of success (Sampaio et al., 2006). This adaptability is evident in fast breaks, where speed enhances scoring opportunities, as well as in more structured plays that aim to maximize possession time to generate higher-quality shot opportunities.

The length of offensive plays and the physical exertion involved are closely linked to the physiological demands players face, influencing the type of metabolic pathways used for energy production. Offensive actions can vary from few seconds to the 24-s shot clock limit, requiring different types of exertion depending on the game’s context and phase (Fox et al., 2019; Pérez-Chao et al., 2023).

Recent research highlights that the basketball’s intermittent nature demands a combination of anaerobic and aerobic energy systems to support intense, short bursts of activity followed by brief recovery periods (Petway et al., 2020). Alactic anaerobic metabolism is predominant in short, explosive actions such as sprints and jumps, while lactic anaerobic and aerobic systems are more engaged during prolonged efforts and during recovery phases between plays (Stojanović et al., 2018). Rapid energy generation through the alactic system is critical in the early quarters of a game, when intensity is the highest, and players are the freshest. As the game progresses and fatigue sets in, the body increasingly relies on the aerobic metabolism to sustain continuous efforts, especially in longer offensive plays and during defensive transitions. Furthermore, managing the duration of offensive actions is closely tied to coaches’ ability to deploy strategies that exploit the opposing team's weaknesses. Serna et al. (2021) observed in the ACB League that teams employing a mix of quick possessions and extended plays, depending on their opponent’s physical condition and response capacity, tended to reduce turnovers and increase shooting accuracy.

Numerous studies have identified a crucial link between play duration and offensive performance (Sosa-Martin et al., 2024). Analysis of elite competitions such as the NBA reveals that effective management of possession time is vital for controlling the game’s rhythm and increasing the likelihood of success (Gonçalves et al., 2022). Teams are often categorized based on their preference for fast possessions (under 10 s) or more deliberate plays (over 20 s), with the choice depending on the game situation (Sirnik et al., 2022). In critical moments, such as the final minutes of a game, possession duration significantly influences shot quality and the ability to execute effective defensive strategies against opponents (Chen et al., 2021).

The length of possessions also has a bearing on a team’s ability to cope with psychological and physical pressures, such as fatigue and stress caused by aggressive defensive play. Fatigue is a key factor in offensive performance, closely related to the game quarter in which actions occur. Research indicates that physical performance and shooting efficiency typically decline in later quarters due to cumulative fatigue (Edwards et al., 2018; Zając et al., 2023). This decline is linked to muscle fatigue and reduced recovery capacity, which notably affects the ability to execute high-intensity actions, such as driving to the basket or making long-range shots (Li et al., 2021).

Research on the game tempo indicates that longer possession duration can alleviate pressure in crucial moments by allowing better offensive organization and more deliberate decision-making (Foteinakis et al., 2024). In leagues such as the NBA, teams that maintain longer possessions in the closing stages of games are more successful at holding leads or overcoming deficits, underscoring the importance of strategic possession management (Zhang et al., 2019).

Advances in observational methodology have enabled more precise analysis of how offensive duration affects team performance. Technologies such as player tracking and data analysis through machine learning allow researchers to break down each play into measurable variables, including player speed and shot efficiency (Gou and Zhang, 2022; Torres-Ronda et al., 2022). These methods have proven valuable not only for analyzing offensive success, but also for identifying optimal patterns of play based on the opponent and game conditions (Fox et al., 2019), as demonstrated in the NBA, where physical defenses tend to extend possession time (Courel-Ibáñez et al., 2018).

It is worth noting that technological advancements and data-driven analysis benefit not only elite teams, but also coaches and analysts at lower levels. Access to precise data on possession duration and offensive efficiency provides teams with a competitive edge, helping to enhance their overall performance. By integrating tools such as video analysis and player tracking systems, teams can adjust their tactics and develop more comprehensive game plans, contributing to a shift in how offense is conceived in modern basketball (Esteves et al., 2021; Zhang et al., 2019). This growing emphasis on possession duration highlights its critical role in shaping strategy and performance in the sport.

This study aimed to compare the offensive duration and shooting effectiveness of the only two NBA teams to achieve the highest number of regular-season victories (82 games), but with different outcomes in the Playoff Finals. The teams were the Chicago Bulls in the 1995/1996 season (72 wins, 10 losses), who won the finals (4–2), and the Golden State Warriors in the 2015/2016 season (73 wins, 9 losses), who lost the finals (3–4).

## Methods

### Research Design

The research employed observational methodology (Anguera, 1979), structured as a Nomothetic, Point, and Multidimensional design (N/P/M) according to Anguera et al. (2011). The study is nomothetic as it analyzed offensive actions of two teams, allowing for generalization based on a wide range of observations. It is point-based, focusing on two specific timeframes: the 1995/1996 NBA Finals for the Chicago Bulls and the 2015/2016 NBA Finals for the Golden State Warriors. Finally, it is multidimensional, as multiple dimensions and categories of offensive plays were analyzed within the observational instrument.

This comprehensive approach enabled a detailed comparison between teams from different eras and playing styles, capturing both differences and similarities in their offensive patterns (Anguera, 2003; Bakeman and Quera, 2011).

### Participants

The sample consisted of all offensive plays executed by the Chicago Bulls during the 1995/1996 playoff finals and by the Golden State Warriors during the 2015/2016 playoff finals. Free-throw actions were excluded, as they are not considered part of active offensive sequences. The sample comprised 1,461 offensive actions, with 669 by the Chicago Bulls across the six playoff games and 792 by the Golden State Warriors across seven playoff games, providing a robust dataset for identifying relevant tactical patterns.

### Instrument and Data Recording Procedure

The study was conducted in accordance with the ethical principles of the Declaration of Helsinki (Bošnjak, 2001; Tyebkhan, 2003; WMA, 2022), which outlines ethical guidelines for research involving human subjects. In this case, no ethical approval was required, as the data were derived from publicly available images.

### Data Recording

Offensive actions were recorded using Lince Plus software (Soto et al., 2022). Lince Plus is designed for coding and analyzing observational data and enables precise segmentation of plays, facilitating subsequent analysis. It is widely recognized for its versatility and reliability in analyzing behaviors in complex sports scenarios (Soto et al., 2022).

The instrument developed by Cook and Amatria (2024) was utilized for the analysis of offensive actions in basketball. Data collection was conducted by two observers, both with degrees in Physical Activity and Sports Sciences and with at least 10 years of experience in the sport under study. Observer training was carried out in accordance with Anguera's (2003) recommendations, ensuring that both shared the same criteria for coding offensive actions. This training process is fundamental in observational methodology to minimize potential biases and ensure homogeneity in the recorded data. When both observers achieved an agreement rate (Cohen, 1960) higher than 0.8 (Landis and Koch, 1977), training for data collection was considered complete.

It is noteworthy that, by taking advantage of the capabilities offered by Lince Plus software, it was possible to capture the dimension of play duration, which underlies the recording of each observed play. Thus, following the classifications developed by Gómez et al. (2016), Oliver (2011), Sampaio et al. (2006), and García-Rubio et al. (2015), the dimension “Type of Play” was established, comprising specific categories based on the duration of the offensive action developed (Table 1).

**Table 1:** Dimension: Type of play and its categories.

Dimension	Category	Code	Description
Play type	Ultra Fast	UF	Plays lasting between 0.01 and 4.00 s
	Fast	F	Plays lasting between 4.01 and 8.00 s
	Semi Fast	SF	Plays lasting between 8.01 and 12.00 s
	Medium	M	Plays lasting between 12.01 and 16.00 s
	Slow	S	Plays lasting between 16.01 and 20.00 s
	Very Slow	MS	Plays lasting longer than 20.00 s

### Data Quality Control

To ensure the reliability of the data, the Cohen's Kappa coefficient (Cohen, 1960) was used, measuring the degree of agreement between two observers, adjusted for the probability of chance agreement. After the training period in which intra-observer agreement was achieved, inter-observer agreement was assessed with the records comprising the sample. GSEQ software, version 5.1, was used to calculate this coefficient, following guidelines established by Bakeman and Quera (2011). In this study, a Kappa coefficient of 0.85 was obtained, reflecting substantial agreement between observers, according to the interpretation standards proposed by Landis and Koch (1977). This level of agreement ensures the validity and reliability of the data collected for subsequent analysis.

### Data Analysis

Statistical analysis of the data was conducted using the Pearson's Chi-square (X^2^) test, which allowed the assessment of significant differences between the offensive strategies of the 1995/1996 Chicago Bulls and the 2015/2016 Golden State Warriors. The Chi-square test enables analysis of categorical data and comparison of the frequency of certain events (González and Pérez de Vargas, 2009), in this case, the type of plays or the duration of possessions.

## Results

The results show that of the 1,461 plays analyzed, 45.79% (669 plays) corresponded to the Chicago Bulls (Bulls) during the six games of the 1995/1996 season playoff finals, averaging between 111 and 112 offensive plays per game. Meanwhile, 54.21% (792 plays) corresponded to the Golden State Warriors (Warriors) over the seven games played during the 2015/2016 playoff finals, with an average of 113 offensive plays per game.

Table 2 presents the results of the analysis of the play typology (ultra fast, fast, semi-fast, medium, slow, and very slow) for each team. It can be observed that the Warriors developed a higher percentage of fast plays (lasting 4–8 s) with 21.6%, and semi-fast plays (lasting 8–12 s) with 30%, compared to the Bulls, whose highest percentages were in the development of medium plays (12–16 s) with 24.2% and semi-fast plays with 21.5%. Significant differences were found (χ^2^ = 38.262, df = 5; *p* < 0.001) when comparing the team and the type of play developed according to its duration.

**Table 2:** Analysis of the team and offensive play typology based on duration.

	Play typology based on duration					
	Ultra Fast	Fast	Semi Fast	Medium	Slow	Very Slow
Bulls	9.0%	16.3%	21.5%	24.2%	16.4%	12.6%
Warriors	10.5%	21.6%	30.4%	18.4%	11.4%	7.7%

Regarding plays that ended in a shot attempt, Table 3 presents results of the analysis between the team and the completion of offensive actions in a shot attempt. It can be seen that the Bulls achieved the highest percentage of shot attempts in plays with medium duration (12–16 s) with 24.1%, while the Warriors achieved their highest percentage in semi-fast plays (8–12 s) with 30%. Significant differences were found (χ^2^ = 30.384, df = 5; *p* < 0.001) when comparing the team and the type of play developed based on the duration of those ending in a shot attempt.

**Table 3:** Analysis of the team and offensive play typology based on duration for plays ending in a shot attempt.

	Play typology based on duration for plays ending in a shot attempt					
	Ultra Fast	Fast	Semi Fast	Medium	Slow	Very Slow
Bulls	9.1%	13.0%	21.5%	24.1%	18.2%	14.1%
Warriors	9.1%	19.8%	30.7%	19.1%	12.6%	8.6%

Table 4 presents the results of the analysis of the success rate of plays ending in a shot attempt. It can be observed that the percentages were similar for both teams, with a slightly higher success rate for the Golden State Warriors (42.1%) compared to the Chicago Bulls (40.1%). No significant differences were found in this analysis.

**Table 4:** Analysis of the success of shots attempted in offensive actions.

	Successful Attempt	
	Unsuccessful	Successful
Bulls	59.9%	40.1%
Warriors	57.9%	42.1%

When analyzing the score obtained from shot attempts, Table 5 shows that the highest percentage for both teams resulted from missed shots, with 59.9% for the Chicago Bulls and 58% for the Golden State Warriors. However, it is important to highlight the high percentage achieved by the Warriors in successful three-point shots, with 16.3%, compared to 8% for the Chicago Bulls. Significant differences were found (χ^2^ = 17.820, df = 2; *p* < 0.001) when comparing the team and the score obtained based on play duration.

**Table 5:** Analysis of the team and the score obtained from plays ending in a shot attempt.

	Basketball Shot Score		
	0 points	2 points	3 points
Bulls	59.9%	32.1%	8.0%
Warriors	58.0%	25.7%	16.3%

**Figure 1:**
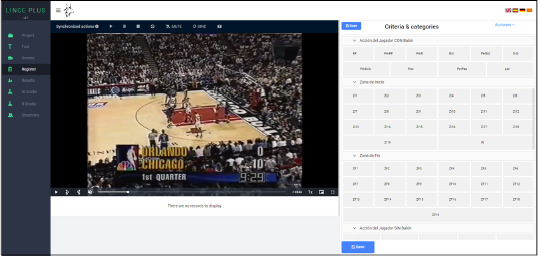
Moment of recording using Lince Plus software (Soto et al., 2019).

## Discussion

This study aimed to compare the offensive duration and shooting effectiveness of the only two NBA teams (i.e., the Chicago Bulls and the Golden State Warriors) to achieve the highest number of regular-season victories (82 games), but with different outcomes in the Playoff Finals. First, it is important to note that despite the passage of time and the observed increase in the average number of offensive plays per game, both teams were very close in this regard, with 111–112 actions for the Bulls and 113 for the Warriors. These results differ from those reported by Cavarkapa et al. (2022) who, in their NBA study, estimated the average number of offensive plays to be between 95 and 105. This result can be explained by the interpretation of offensive actions in this study, as the systematic recording method used by the authors considered offensive rebounds, often viewed as a continuation of possession, as new offensive actions due to the additional scoring opportunity.

In terms of the play typology, significant differences were found between the two teams. In this case, the difference in the playing style was evident, with the Bulls developing plays of longer duration compared to the Warriors. These results align with those of Sampaio and Janeira (2003) who argue that a higher tempo generates more scoring opportunities, which was evident in the dynamics of the Warriors. These findings are also supported by Falcão et al. (2015) who highlight that the Bulls' offensive game was influenced by coach Phil Jackson's “magic triangle”, resulting in longer plays.

In this regard, the Bulls demonstrated stability in their play development, with similar percentages across fast, semi-fast, medium, and slow plays, indicating a varied rhythm and a combination of offensive strategies that reflected tactical variability and flexibility. In contrast, Golden State's play was concentrated on actions lasting less than 12 s, making their gameplay more predictable. The lack of variability, along with their results, underscores the importance of tactical flexibility, as offensive success depends not only on speed, but also on the ability to adjust possession to match the context of the game and the team's needs at specific moments (Romaris et al., 2012).

From the perspective of a team's ability to respond to psychological and physical pressures, such as fatigue and the stress of the opponent’s defense, it is clear that the Chicago Bulls employed longer plays, resulting in better management of critical game situations (Cabarkapa et al., 2022). These results are consistent with Mandić et al. (2019) who argued that in top-level professional leagues (NBA and Euroleague), teams that sustained longer possessions in the final stages of games tended to have more success in maintaining leads or making comebacks, highlighting the strategic importance of time management in offensive possessions.

Regarding the effectiveness of shot attempts, both teams had similar success rates. However, when looking deeper into scoring efficiency, significant differences were found in the value of successful shots, particularly in the higher success rate of three-point shots by the Golden State Warriors compared to two-point shots by the Chicago Bulls. This increased success in three-point shooting is in line with findings from Hu (2024) who emphasizes the impact of three-point shot accuracy on the offensive strategies of high-level basketball teams.

Combining this effectiveness with the aforementioned tempo of play reinforces the shift in the playing style, as Sampaio and Janeira (2003) noted, where a faster pace generates more scoring opportunities, but also wears down opposing defenses through repeated high-intensity efforts.

## Conclusions

The objective of this study was to compare the duration of plays and the shooting effectiveness of the only two NBA teams to achieve the most wins in a season: the Chicago Bulls in 1995/1996 and the Golden State Warriors in 2015/2016. Notably, the Bulls won their series while the Warriors were defeated.

The comparative analysis of offensive actions revealed significant differences in the pace of execution, with the Warriors favoring shorter, more explosive plays, while the Bulls developed more deliberate and strategic actions. Despite these differences, shooting effectiveness was similar between the two teams. The number of offensive plays per game was also close, suggesting an evolution in the playing style over time, with both teams achieving historic success despite different approaches.

The practical implications of this research are of particular interest to coaches and analysts seeking to optimize offensive strategies in basketball. Understanding the tempo and play duration that lead to scoring opportunities, as well as the value of shot success, offers valuable insights for planning offensive tactics based on a team’s specific strengths and weaknesses. For example, teams aiming to capitalize on speed may focus on fast-break opportunities and quick ball movement, while those favoring a slower tempo might develop structured offensive sets that maximize shot quality.

In terms of limitations, the study’s scope was restricted to the finals of both seasons, potentially omitting variations in the playing style and effectiveness observed during regular-season games. Future research could expand this analysis to include different stages of the season, as well as the impact of defensive strategies on offensive play duration and success. Additionally, the influence of individual players on team dynamics and the play tempo could be explored, given the unique contributions of key figures like Michael Jordan and Stephen Curry to their respective teams.

By continuing to investigate these factors, further insights can be gained into the evolving nature of basketball tactics and the interplay between pace, shot success, and overall team performance.
